# Religious Coping, Religiosity, Depression and Anxiety among Medical Students in a Multi-Religious Setting

**DOI:** 10.3390/ijerph16020259

**Published:** 2019-01-17

**Authors:** Benedict Francis, Jesjeet Singh Gill, Ng Yit Han, Chiara Francine Petrus, Fatin Liyana Azhar, Zuraida Ahmad Sabki, Mas Ayu Said, Koh Ong Hui, Ng Chong Guan, Ahmad Hatim Sulaiman

**Affiliations:** 1 Department of Psychological Medicine, Faculty of Medicine, University of Malaya, Kuala Lumpur 50603, Malaysia; jesjeet@um.edu.my (J.S.G.); efel_fla@yahoo.com (F.L.A.); zuraidaas@ummc.edu.my (Z.A.S.); owenfowler@yahoo.com (K.O.H.); chong_guan@um.edu.my (N.C.G.); hatim@um.edu.my (A.H.S.); 2 Department of Parasitology, Faculty of Medicine, University of Malaya, Kuala Lumpur 50603, Malaysia; hanzhai2574@gmail.com; 3 Hospital Raja Permaisuri Bainun, Jalan Raja Ashman Shah, Ipoh 30450, Malaysia; chiarafrancinepetrus@gmail.com; 4 Department of Social and Preventive Medicine, Faculty of Medicine, University of Malaya, Kuala Lumpur 50603, Malaysia; mas_ayu@um.edu.my; 5 Julius Centre University of Malaya, Faculty of Medicine, University of Malaya, Kuala Lumpur 50603, Malaysia; 6 University of Malaya Centre of Addiction Sciences, Faculty of Medicine, University of Malaya, Kuala Lumpur 50603, Malaysia

**Keywords:** coping, mental health, anxiety, depression, medical students

## Abstract

Medical students are vulnerable to depression and anxiety due to the nature of their academic life. This study aimed to determine the prevalence of depressive and anxiety symptoms among medical students and the association between religious coping, religiosity and socio-demographic factors with anxiety and depressive symptoms. A cross sectional design was used for this study. Scales used were the Malay version of the Duke Religious Index (DUREL-M), the Malay version of the Brief Religious Coping Scale (Brief RCOPE) and the Malay version Hospital and Anxiety Depression Scale (HADS-M). 622 students participated in this study. They scored moderately on the organized (mean: 3.51) and non-organized religious (mean: 3.85) subscales of the DUREL, but had high intrinsic religiosity (mean: 12.18). The prevalence of anxiety and depressive symptoms were 4.7% and 17.4% respectively, which is lower than local as well as international data. Islam, negative religious coping and the presence of depressive symptoms were significantly associated with anxiety symptoms. Only the presence of anxiety symptoms was significantly associated with depressive symptoms. Negative religious coping, rather than positive religious coping, has significant association with depressive and anxiety symptoms. Redirecting focus towards negative religious coping is imperative to boost mental health outcomes among medical students.

## 1. Introduction

Depression and anxiety are common mental illnesses that are often overlooked amongst medical students. Recent studies concluded that the global prevalence of depression or depressive symptoms among medical students ranged from 27% to 34.6% [1,2,3], which are staggeringly high figures. Anxiety symptoms, though not as widely studied as depression, were shown to be as prevalent, with figures between 35% and 63% [3,4,5]. The impact of untreated mental illness on these students’ performance during their most formative years in medical school cannot be overstated. Several factors have been linked to increased adverse mental health effects in medical students. A recent meta-analysis found that pre-clinical years and staying at home were risk factors for medical students to develop depression [6,7]. In addition, untreated stress, anxiety and depression have been shown to be risk factors for adverse mental health outcomes and suicidal ideations [8]. Given that the South East Asian region is the proverbial melting pot for many religions and cultures [9], it is interesting to ascertain the role of religious coping in this particular group.

Religion and mental health have been generally viewed as allies, with positive religious coping providing much needed solace in times of distress [10,11]. Religious coping, as described by Pargament, refers to seeking religion as a means of strength in difficult times and includes reading holy scriptures, seeking counsel from religious leaders and decreasing distress or unpleasant thoughts using religious means [12]. It can be further divided into two constructs, which are positive and negative religious coping. Positive religious coping translates to a secure relationship with God, and involves appraisal of obstacles in the light of God’s providence. Negative religious coping, on the other hand, is maladaptive and interprets challenges as a result of punishment and divine dissent [13]. Positive religious coping, however, has been shown to be less impactful on mental health as several studies have proved that negative religious coping had more merit in predicting anxiety and depression compared to positive religious coping [11,14,15].

The relationship between religion and mental health is not a simple one. The landmark review by Koenig and colleagues summarized that a majority of studies favored a positive relationship between religion and mental health [16]. More recent studies however have had varied results. In particular, the concept of religious strain was introduced to explain the phenomena whereby religiosity can lead to a strenuous relationship with God and as a result lead to a decline in coping and mental health as a whole [17,18]. A study among an Iranian cohort of medical students found that religiosity negatively predicted depression and anxiety [19]. Similarly, another study involving Muslim medical students in Iran showed that religiosity was protective against depression [20]. However, another study among Jewish medical students found that there was no significant association between religiosity, depression and anxiety [21]. In view of these mixed results, there is a need for more studies in this area to further define the relationship between religion and mental health.

This study was conducted in Malaysia, a country with a pluralistic society, with Islam as the national religion. However, Christianity, Hinduism, Buddhism, Taoism and other religions are practiced freely. Given the multi-ethnic and multi-religious background in Malaysia, it is important to ascertain the effect of ethnicity and religion on adverse psychiatric events. In such a diverse population, the Islamic religion in particular, was found to be an important factor that protected the Malay people from adverse mental health outcomes including suicide attempts [22]. Importantly, prior studies on the subject of religiosity and mental health centered on populations with a single-religion majority population [10]. No prior studies have been conducted to assess the relationship between religious coping, depression and anxiety among medical students in Malaysia, even though religion is an important part of the social fabric in this region [23].

The primary objective of this study was to examine the correlation between religious coping, religiosity, depressive and anxiety symptoms among medical students in a pluralistic, multi-religious Malaysian setting. The secondary objective was to determine the prevalence and association of depressive and anxiety symptoms among the medical students studied.

## 2. Materials and Methods

This was a cross sectional study done among Year one to Year five undergraduate medical students at the Faculty of Medicine, University of Malaya. University of Malaya is the nation’s premier institute of higher learning situated at the heart of Kuala Lumpur, and consists of students from different cultural and religious backgrounds. A non-probabilistic convenience sampling method was employed and students who consented to the study were given questionnaires to answer. Those individuals who agreed to join the study were screened according to the following inclusion and exclusion criteria. Out of 697 medical students, 622 students who fulfilled the inclusion criteria and did not meet the exclusion criteria participated in the study.

### 2.1. Inclusion Criteria

- Malaysian medical students currently pursuing studies in their medical degree at the University of Malaya.
- Aged 19 and above.
- Students must be in their first degree programs.

### 2.2. Exclusion Criteria

- Illicit substance usage.
- Refusal to participate in study.

Socio-demographic information was collected via pre-designed questionnaires made for the study. Religiosity was measured using the Duke Religious Index Malay Version (DUREL-M) whereas religious coping was measured using the Malay Version of the Brief Religious Coping Scale (Brief RCOPE-M). Depressive and anxiety symptoms were measured using the Malay Version of the Hospital Anxiety and Depressive Scale (HADS-M). All scales were validated for use in the Malaysian population. The Medical Ethical Committee of University Malaya Medical Centre approved the study (MREC 20175275274).

### 2.3. Measurement Tools

#### 2.3.1. DUREL

This scale was used to measure religiosity in the study population. It is a brief measure of religiosity consisting of five items that measure three dimensions of religiosity: Organized Religious Activity (ORA), Non-Organized Religious Activity (NORA) and Intrinsic Religiosity (IR) [24]. ORA refers to communal religious activities such as attending public places of worship and religious activities. NORA refers to religious activities conducted in a personal manner, such as private scripture reading, and personal prayer time. IR assesses the degree of personal religious commitment and motivation. The DUREL was translated to the Malay language as the DUREL-M (Duke Religious Index Malay Version) and has been validated in the Malaysian population. It scores between a range of 5–27. The translated version had a good internal reliability of 0.8 [25].

#### 2.3.2. HADS

The HADS was used to screen for anxiety and depressive symptoms in the study population. It has been widely used among the general population including the medical student population [26,27,28]. This scale consists of 14 items and has been validated in the local setting as the Hospital Anxiety and Depression Scale Malay Version (HADS-M). A lower cut-off score of 8 or 9 was found to be appropriate for the Malaysian population as it had a sensitivity of 93.2% and specificity of 90.8%. The translated scale was shown to have a Cronbach’s alpha of 0.87 [29]. Choosing the more conventional cut-off point of 11 would have missed out a significant proportion of Malaysians with anxiety and depressive symptoms [30]. In this study, the cut-off of 8 was chosen for the HADS-M.

#### 2.3.3. Brief RCOPE

This scale is a brief tool to assess religious coping, which consists of 14 items. It consists of seven positive coping items and seven negative coping items and was developed by Pargament to assess the role of religion in coping with challenges and stresses in life. It consists of positive coping and negative coping methods. Positive coping methods refer to harnessing a positive relationship with God as a means of coping and involves praying, meditating and reflecting on God to help in times of distress. Negative coping methods occur when one blames God for their mishaps and believes the trauma or challenges being faced is punishment from God [31]. This scale was translated into the Malay language with a Cronbach’s alpha of 0.87 for positive coping (*P* COPE) and 0.88 for negative coping (*N* COPE) [32].

### 2.4. Statistical Analyses

The data in this study were analyzed using the Statistical Package for Social Science version 25.0 (IBM Corp., Armonk, NY, USA). Descriptive statistics were carried out to summarize the characteristics of the participants. Mean and standard deviations were used to report continuous typed variables whereas categorical typed variables were presented by frequency followed by percentage. Religious typed variables were further correlated with anxiety and depressive symptoms through Spearman rank correlation analysis. Bivariate analysis was used to investigate the risk factors (socio demographic, psychiatric factors and religious typed factors) associated with the increment of anxiety and depression. Factors with *p* value less than 0.05 were subjected to multivariate analysis.

## 3. Results

### 3.1. Socio-Demographic Data

The first table illustrates the socio-demographic data of the study population. A total of 622 out of 697 medical students took part in this study, giving the participation rate of 89%. Year one and year five medical students were the majority of the participants (23.6% and 24% respectively), whereas there were similar representations between years two until four. More female (64.8%) than male (35.2%) students participated in the study and most of them were not married (81.5%). Islam and Buddhism were the dominant religions, with percentages of 46.1% and 29.9%, respectively. Almost all of them (98.9%) were full-time students and the majority came from middle to high-income families (69.8%). As for student living, 96.3% of students stayed in hostel accommodation, either alone (38.9%) or with roommates (57.4%). With regards to university related payments, government or private scholarships/loans covered more than half of the students’ fees. 4.7% of the students experienced anxiety symptoms whereas 17.4% of them exhibited depressive symptoms with 15.9 % reporting symptoms of both disorders (Table 1).

Anxiety symptoms were shown to increase with year of study, and peaked at year 5 (21.5%). For depressive symptoms, a similar trend was seen whereby depressive symptoms increased with year of study with the exception of year 5 medical students, whereby the rates of depressive symptoms declined to 2.7%. Year 3 medical students expressed the highest rates of mixed anxiety and depressive symptoms. Generally, anxiety and depressive symptoms increased with year of study however reached a plateau during the last two senior years (years 4 and 5) (Table 2).

Interestingly, mixed symptoms were higher among year 1–3 medical students compared to anxiety or depressive symptoms alone, however lower compared to anxiety symptoms between year 4 and 5 medical students (Table 2). Regarding severity of depressive and anxiety symptoms, majority of students who were anxious or depressed were in the mild category (19.5 % and 21.9% respectively). Only a minor percentage of students had severe symptoms. 3.2% reported severe anxiety symptoms whereas 0.3 % exhibited severe depressive symptoms (Table 3).

Our study reported higher mean scores of depressive symptoms compared to other studies done. On the other hand, the mean anxiety scores from our study was lower than other previously published studies. The one sample *t*-test conducted showed that the mean difference between the results were significant, except for the differences between the HADS-D score in the Turkish study with our mean score (Table 4).

### 3.2. Religiosity, Religious Coping and Its Correlation with Depressive and Anxiety Symptoms

On average, the medical students who participated in this study were fairly religious. They attended religious related activities a few times annually, with a mean ORA score of 3.51 (maximum score of 6). In addition, medical students engaged in non-organized religious activities such as reading holy scriptures or personal prayer at least two times per week as shown by the mean NORA score of approximately 4.00 (maximum score of 6). Additionally, the IR scores among the students were relatively high (12.18 out of a maximum score of 15.00). The total mean DUREL-M score was 19.54 out of a possible maximum score of 27. The medical students showed more positive compared to negative religious coping, as evidenced by a mean score of 19.81 vs. 10.16 respectively (Table 5). These results indicate that the medical students who participated in the study relied more on positive coping, rather than relying on negative coping. Spearman rank correlation analysis revealed only negative religious coping was significantly (*p* < 0.01) correlated with both anxiety (ρ = 0.183) and depressive symptoms (ρ = 0.118) of the students. In terms of religiosity, only intrinsic religiosity (IR) was significantly correlated with depressive symptoms (ρ = −0.087) but the effect sizes of these associations were very small (Table 5). Positive coping was significantly correlated with all measures of religiosity, however negative coping was not.

### 3.3. Univariate and Multivariate Analyses for the Correlates of Anxiety Symptoms

Univariate General Linear Model (GLM) analysis was done to assess the correlates of anxiety symptoms. It revealed that current year of study, religion, staying alone, negative religious coping as well as depressive symptoms, were significantly (*p* < 0.05) associated with anxiety (Table 6). After adjusting for multivariate through GLM, only religion (Islam), negative religious coping and depressive symptoms remained significant. Among these three variables, depression showed the highest estimate effect size (0.479), followed by negative religious coping (0.038). The effect size of Islam on anxiety was very small at 0.008. Other socio-demographic factors were not significantly associated with anxiety symptoms. The multivariate GLM showed that Islam, negative religious coping and depressive symptoms were associated with higher anxiety symptoms (Table 6).

### 3.4. Univariate and Multivariate Analyses for the Correlates of Depressive Symptoms

As for depressive symptoms, univariate GLM revealed that only negative religious coping and anxiety symptoms were significantly correlated (Table 7). The univariate analysis showed that negative coping had an estimated effect size of 0.012 in relation to depressive symptoms. This showed that students with negative coping were correlated with higher depressive symptoms, though with a relatively small effect size. Of these two factors, multivariate GLM showed that only anxiety symptoms were significantly associated with depression with an estimated effect size of 0.475. Religiosity and religious coping were not significantly associated with depressive symptoms among medical students.

## 4. Discussion

Mental illness among medical students has often been swept under the carpet and under-recognized [34], though the rates of these mental illness among this vulnerable population are by no means trivial. A recent meta-analysis of depression among medical students concluded that the global prevalence was around 28% [1]. The prevalence of depressive and anxiety symptoms in our study was found to be approximately 17% and 5% respectively. Studies done abroad summarized that the prevalence of depression and anxiety among medical students ranged between 1.4–73.5% [2] and 28–85% [35,36]. Interestingly, the anxiety and depression prevalence data from this study was lower than global data. One possible reason is due to the paternalistic and hierarchical nature of medicine practiced in the Asian region [37], mental illness is often perceived to be a sign of weakness, and thus students may have under-reported their symptoms. Given that the samples were obtained from the nation’s premier medical institution, the medical students who participated could also have been more resilient and have better coping mechanisms compared to other medical students, though this hypothesis needs more evidence in the form of well-designed studies.

A majority of the students showed mild symptomatology, and only a minority of them was in the severe bracket. Our results differ from another Malaysian study that concluded most medical students showed moderate, followed by severe symptoms of anxiety and depression [5]. The results in the current study population provide fertile grounds for early intervention before medical students slip into more severe symptomatology. The mean score of depression in our study (Table 4) was higher than the mean scores in similar study populations internationally [26,27,28]. Higher scores of depression in our sample could be attributed to the particularly stressful exam-oriented environment prevalent among Malaysian medical students, who perceive academic excellence as the marker of a sound and safe doctor. The medical students in University Malaya, where this study was conducted, also used a newly adapted academic syllabus known as the University of Malaya Medical Program. Thus they could still be adjusting to the rigors and challenges of this new learning module. Malaysian medical students however fared better in anxiety compared to their international counterparts and undergraduates who took other courses [27,33]. As anxiety symptoms were correlated to depressive symptoms in our study (Table 7), reduced anxiety in our population may be protective against the development of more severe mood disorders in our population.

The only sociodemographic factor found to be associated with anxiety was the Islamic religion. However, the effect size was very small (η^2^: 0.008) and this outcome can be explained by the presence of social stigma, which is especially strong within the Muslim community [38], increasing anxiety symptoms in this population. Our study did not find a markedly significant association between religiosity, anxiety and depressive symptoms among the medical student population. This finding is similar to an Israeli study done among medical students, which showed that religiosity was not correlated with anxiety and depression [21]. Malaysian medical students showed moderate organized and non-organized religious activity but scored fairly high on intrinsic religiosity. The intrinsic religiosity score in our study (12.18) was higher than a previous study done among Brazilian medical students (mean IR score: 9.63) [39]. Despite this, religiosity was not associated with depressive or anxiety symptoms in our population. There are several possible explanations for the results seen. Our study population was a heterogeneous sample, consisting of students from various cultures and religions. Given the pluralistic and multi-religious nature of the study population, the concept and interpretation of religiosity may be variable and they may subscribe to the concept of spirituality, rather than formal religion. Though spirituality and religiosity do overlap, there are pivotal differences between these two constructs. Spirituality refers to a more personal and individualized interpretation in the search for meaning, whereas religiosity is characterized by the dogmatic and institutionalized interpretation of the sacred [40]. Furthermore, religion is a multi-dimensional construct and religiosity as measured by scales may not be completely reflective of the strength and nature of one’s engagement in religious activities. Thus, scales alone may not be an adequate measure of religiosity in its true sense. In conclusion, our study further bolstered present evidence that religiosity does not necessarily correlate with better or worse depressive and anxiety scores among medical students [21,41].

In this study, the medical students used more positive coping compared to negative religious coping (mean score: 5.99 vs. 3.57). In the multivariate analysis, negative religious coping was associated with increased anxiety but not depressive symptoms, similar to another study conducted among Malaysian psychiatric patients [42] and Somalian college students [43]. Though positive religious coping was correlated with all measures of religiosity, it did not significantly associate with anxiety and depression. The results in our study concurred with other published data [44,45] in concluding that negative religious coping exhibited a stronger effect on mental health outcomes compared to positive religious coping. Negative religious coping is associated with maladaptive emotional regulation and as a result gives rise to dysphoric and distressing emotional states, paving the way for mood disorders [44]. Thus, it is not surprising that this form of religious coping is associated with increased anxiety in our study population. Our results thus add to the body of evidence that negative religious coping had more merit compared to positive religious coping in modulating mental health issues.

Our study is not devoid of limitations. The cross-sectional nature of this study rendered it unable to assess causality of the factors, and can at best study their association. This study was done in a single public university and did not take into account other institutions. Including other public and private universities would have enhanced generalizability of the results from this study. Since both anxiety and depression were strongly correlated with each other (η^2^: 0.479 and η^2^: 0.475 respectively), future studies should analyze potential mediating factors between these two illnesses and the role of religious coping and religiosity in this context. Another important limitation was that stress as a potential confounder to anxiety and depression was not taken into account in this study. Inclusion of stress could have added newer dimension to the relationship between the variables studied.

## 5. Conclusions

To the authors’ knowledge, this is the first study done in the South East Asian region to assess the association between religious coping, religiosity, depression and anxiety amongst medical students. In conclusion, the prevalence of depressive and anxiety symptoms among medical students in a local university in Malaysia were lower than previous local and international studies done, reflecting perhaps increased resilience in this population. Similar to other populations studied, our data showed that negative, rather than positive religious coping, had stronger association with adverse mental health outcomes among medical students. Improving negative religious coping by means of psycho-education and religious cognitive restructuring may be the panacea to lesser psychiatric issues among this vulnerable population. Despite the nuances of a multi-religious society in Malaysia, it is imperative to identify and improve maladaptive religious coping in order to boost mental health among medical students. Future studies should look at the mediation between religion, spirituality and mental health outcomes to further study the relationship between these variables.

## Figures and Tables

**Table 1:** Socio-demographic characteristics and mental health status of medical students (*n* = 622).

Socio-Demographic	Mean (SD)	*n* (%)
Age	21.18 (1.53)	
Gender		
Male		219 (35.2)
Female		403 (64.8)
Year of study		
1		147 (23.6)
2		102 (16.4)
3		114 (18.3)
4		110 (17.7)
5		149 (24.0)
Family income (RM)		
Low (<2300)		132 (21.2)
Middle (2300–5599)		245 (34.9)
High (>5600)		245 (34.9)
Religion ^†^		
Islam		287 (46.1)
Christianity		59 (9.5)
Hinduism		53 (8.5)
Buddhism		186 (29.9)
Taoism		20 (3.2)
Others		17 (2.7)
Relationship status ^†^		
Single		507 (81.5)
In a relationship		112 (18.0)
Married		3 (0.5)
Pre-University qualification ^†^		
Matriculation		408 (65.6)
STPM		7 (1.1)
A Levels		36 (5.8)
Others		171 (27.5)
Family history of depression		
Yes		44 (7.1)
No		578 (92.9)
Concurrent Jobs while Studying		
Yes		7 (1.1)
No		615 (98.9)
Living Arrangements ^†^		
Hostel (Alone)		242 (38.9)
Hostel (With roommate)		357 (57.4)
Living with Family		15 (2.4)
Living in private outside campus (Alone)		1 (0.2)
Living in private outside campus (With roommate)		7 (1.1)
Source of Finance for Studies ^†^		
Own		177 (28.5)
Government Scholarship/Loan		406 (65.3)
Private Scholarship/Loan		39 (6.3)
Student status		
Negative		386 (62.0)
Anxiety		29 (4.7)
Depressive symptoms		108 (17.4)
Anxiety and Depressive symptoms		99 (15.9)

^†^ Some variables were further dummy coded to minimize uneven data distribution in univariate and multivariate analysis: Religion = Islam vs. non-Islam. Relationship status = Single vs. non-single. Pre-University qualification = Matriculation vs. non-Matriculation. Living arrangements = Hostel vs. not staying in hostel; Staying alone vs. not staying alone. Source of Finance for studies = Self vs. scholarship.

**Table 2:** Anxiety, depressive and mixed symptoms of medical students (*n* = 622).

Year of Study	Anxiety Symptoms (%)	Depressive Symptoms (%)	Anxiety and Depressive Symptoms (%)
1	12.2	4.1	12.9
2	12.7	5.9	13.7
3	15.8	6.1	20.2
4.	24.5	5.5	16.4
5.	21.5	2.7	16.8

**Table 3:** Severity of depressive and anxiety symptoms (*n* = 622).

Symptom Severity	Anxiety Symptoms *n* (%)	Depressive Symptoms *n* (%)
Negative (<8)	375 (60.3)	433 (69.6)
Mild (8–10)	121 (19.5)	136 (21.9)
Moderate (11–14)	106 (17.0)	51 (8.2)
Severe (15–21)	20 (3.2)	2 (0.3)

**Table 4:** Comparative mean of anxiety and depressive symptoms among Malaysian medical students with similar studies from other countries.

Previous Studies and Country of Origin	Instrument Used	Sample Size Estimated Mean (E.M)	Current Study’s Sample Size with Estimated Mean Values	Mean Difference (95% CI)	One Sample *t*-Test, *p*-Value
Quince, T.A.; et al [26]—United Kingdom	HADS-D *	Sample size = 725 core science + 364 clinical students	Sample size = 622 (Year 1–5)		
		Core science components	(*n* = 363)		
		Year 1–3 = 3.39	Year 1–3 = 5.92	2.53 (2.22–2.84)	<0.001
		Clinical components	(*n* = 259)		
		Year 4–5 = 2.37	Year 4–5 = 6.12	3.75 (3.39–4.12)	<0.001
Karaoglu, N.; et all [28]—Turkey	HADS-A ** and HADS-D	Sample size = 164 Year 1 + 186 Year 2 medical students	Sample size = 249 (Year 1 and 2)		
		HADS-A = 7.66	HADS-A = 6.29	−1.371 (−1.85–(−0.89))	<0.001
		HADS-D = 5.77	HADS-D = 5.82	0.49 (−0.32–0.41)	0.790
Bunevicius, A.; et all [27]—Lithuania	HADS-A and HADS-D	Sample size = 360 medical + 84 humanities students	Sample size = 622 (Year 1–5)		
		Medical students			
		HADS-A = 7.50	HADS-A = 6.87	−0.63 (−0.94–(−0.32))	<0.001
		HADS-D = 5.00	HADS-D = 6.00	1.01 (0.77–1.24)	<0.001
		Humanities students			
		HADS-A = 8.50	HADS-A = 6.87	−1.63 (−1.94–(−1.32))	<0.001
		HADS-D = 4.90	HADS-D = 6.00	1.11 (0.87–1.34)	<0.001
Matsudaira, T.; et all [33]—Japan	HADS-A and HADS-D	Sample size = 541 undergraduates	Sample size = 622 (Year 1–5)		
		General undergraduates			
		HADS-A = 7.30	HADS-A = 6.87	−0.43 (−0.74–(−0.12))	0.007
		HADS-D = 5.20	HADS-D = 6.00	0.81 (0.57–1.04)	<0.001

* HADS-D: Hospital Anxiety and Depression Scale—Depression; ** HADS-A: Hospital Anxiety and Depression Scale—Anxiety.

**Table 5:** Spearman correlation analyses between religious-type scaled variables, anxiety and depressive symptoms.

	Mean (SD)	ORA (DUREL-M)	NORA (DUREL-M)	IR (DUREL-M)	*P* COPE	*N* COPE	HADS (Anxiety)
ORA (DUREL-M)	3.51 (1.20)	1.000					
NORA (DUREL-M)	3.85 (1.93)	0.366 **	1.000				
IR (DUREL-M)	12.18 (3.12)	0.381 **	0.542 **	1.000			
*P* COPE	19.81 (5.99)	0.272 **	0.560 **	0.650 **	1.000		
*N* COPE	10.16 (3.57)	0.040	0.074	0.042	0.108 **	1.000	
HADS-M (anxiety)	6.87 (3.95)	−0.016	0.064	−0.041	0.042	0.183 **	1.000
HADS-M (depression)	6.00 (2.99)	−0.036	−0.011	−0.087 *	−0.025	0.118 **	0.720 **

SD = standard deviation. * *p* < 0.05. ** *p* < 0.01. DUREL-M: Duke Religious Index Malay Version ORA: Organized Religious Activity NORA: Non-Organized Religious Activity IR: Intrinsic Religiosity *P* COPE: Positive coping *N* COPE: Negative coping HADS-M: Hospital Anxiety and Depression Scale Malay Version.

**Table 6:** General Linear Model on factors associated with anxiety based on (*n* = 622).

Factors/Variables	Univariate-GLM			Multivariate-GLM		
	b ^1^ (95% CI)	*p* Value	Partial eta ^2^	b ^2^ (95% CI)	*p* Value	Partial eta ^2^
Age ^†^	0.315 (0.113–0.517)	**0.002**	**0.015**			
Gender		**0.255**	**0.002**			
Male	0.377 (−0.273–1.028)	0.255	0.002			
Female *						
Year ^#^		**0.038**	**0.016**		**0.320**	**0.008**
1	−1.246 [−2.143–(−0.349)]	0.007	0.012	−0.441 (−1.411–0.529)	0.372	0.001
2	−0.955 (−1.947–0.036)	0.059	0.006	0.130 (−0.866–1.127)	0.797	0.000
3	−0.434 (−1.393–0.526)	0.375	0.001	−0.142 (−1.015–0.731)	0.750	0.000
4	−0.089 (−1.058–0.881)	0.857	0	0.321 (−0.432–1.073)	0.650	0.001
5 *						
Family income		**0.168**	**0.006**			
Low						
Middle	−0.393 (−1.219–0.453)	0.369	0.001			
High *	0.404 (−0.295–1.104)	0.257	0.002			
Islam		**0.030**	**0.008**		**0.031**	**0.008**
Yes	0.688 (0.067–1.310)	0.030	0.008	0.486 (0.045–0.927)	0.031	0.008
No *						
Relationship		**0.497**	**0.001**			
Single	−0.277 (−1.078–0.524)	0.497	0.001			
Non-single *						
Family history Depression		**0.348**	**0.001**			
Yes						
No *	0.580 (−0.632–1.793)	0.348	0.001			
Pre U qualification		**0.845**	**0.000**			
Matriculation						
Non-matric	0.065 (−0.590–0.720)	0.845	0			
Current jobs		**0.917**	**0.000**			
Yes	−0.157 (−3.106–2.792)	0.917	0.000			
No *						
Hostel		**0.237**	**0.002**			
Yes	0.993 (−0.653–2.640)	0.237	0.002			
No *						
Stay alone		**0.005**	**0.013**		**0.202**	**0.003**
Yes	0.916 (0.283–1.550)	0.005	0.013	0.483 (−0.216–1.228)	0.202	0.003
No *						
Academic financial source		**0.769**	**0.000**			
Own						
Scholarship *	0.103 (−0.586–0.792)	0.769	0.000			
ORA	−0.009 (−0.268–0.250)	**0.947**	**0.000**			
NORA	0.147 (−0.015–0.308)	**0.075**	**0.005**			
IR	−0.041 (−0.140–0.059)	**0.425**	**0.001**			
*P* COPE	0.032 (−0.020–0.084)	**0.223**	**0.002**			
*N* COPE	0.235 (0.150–0.321)	**0.000**	**0.045**	0.156 (0.094–0.218)	**0.000**	**0.038**
Depression	1.380 (0.877–1.883)	**0.000**	**0.480**	0.889 (0.815–0.962)	**0.000**	**0.479**

* Reference group. ^†^ Multicollinearity issue: Spearman correlation coefficient between “Age” and “Year” was 0.967 with *p* value significant at the 0.01 level (two-tailed). Therefore, only “Year” variable was involved in multivariate analysis. Variables with *p* value less than 0.05 were retained for multivariate analysis. b ^1^: crude regression coefficient; b ^2^: adjusted regression coefficient. CI: Confidence interval. Partial eta squared: Estimated effect size. Bolded *p* values and partial eta squared indicate comparison inter-variables whereas un-bolded values were comparison groups within the same variable using least square difference (LSD). ORA: Organized Religious Activity NORA: Non-Organized Religious Activity IR: Intrinsic Religiosity *P* COPE: Postive coping; *N* COPE: Negative coping.

**Table 7:** General Linear Model on factors associated to depression based on (*n* = 622).

Factors/Variables	Univariate-GLM			Multivariate-GLM		
	b ^1^ (95% CI)	*p* Value	Partial eta ^2^	b ^2^ (95% CI)	*p* Value	Partial eta ^2^
Age	0.101 (−0.053–0.255)	**0.199**	**0.003**			
Gender		**0.198**	**0.003**			
Male	0.324 (−0.169–0.817)	0.198	0.003			
Female ^†^						
Year		**0.743**	**0.003**			
1	−0.310 (−0.994–0.374)	0.373	0.001			
2	−0.469 (−1.225–0.287)	0.224	0.002			
3	−0.054 (−0.786–0.678)	0.884	0.000			
4	−0.167 (−0.907–0.572)	0.657	0.000			
5 ^†^						
Family income		**0.259**	**0.004**			
Low						
Middle	0.343 (−0.291–0.977)	0.289	0.002			
High ^†^	0.429 (−0.102–0.959)	0.113	0.004			
Islam		**0.562**	**0.001**			
Yes	0.140 (−0.333–0.613)	0.562	0.001			
No ^†^						
Relationship		**0.308**	**0.002**			
Single	0.315 (−0.291–0.922)	0.308	0.002			
Non-single ^†^						
Family history Depression		**0.108**	**0.000**			
Yes						
No ^†^	0.753 (−0.165–1.671)	0.108	0.000			
Pre-U qualification		**0.150**	**0.003**			
Matriculation	0.364 (−0.132–0.859)	0.150	0.003			
Non-matric ^†^						
Current jobs		**0.312**	**0.002**			
Yes	1.151 (−1.082–3.384)	0.312	0.002			
No ^†^						
Hostel		**0.231**	**0.002**			
Yes	−0.763 (−2.010–0.485)	0.231	0.002			
No ^†^						
Stay alone		**0.368**	**0.001**			
Yes	0.222 (−0.261–0.705)	0.368	0.001			
No ^†^						
Study financial source		**0.245**	**0.002**			
Own						
Scholarship ^†^	0.309 (−0.213–0.831)	0.245	0.002			
ORA	−0.030 (−0.226–0.167)	**0.767**	**0.000**			
NORA	−0.010 (−0.133–0.112)	**0.870**	**0.000**			
IR	−0.074 (−0.149–0.002)	**0.055**	**0.006**			
*P* COPE	−0.014 (−0.053–0.026)	**0.491**	**0.001**			
*N* COPE	0.092 (0.027–0.158)	**0.006**	**0.012**	−0.33 (−0.082–0.016)	**0.186**	**0.003**
Anxiety	0.525 (−0.482–0.568)	**0.000**	**0.480**	0.531 (0.487–0.575)	**0.000**	**0.475**

^†^ Reference group. Variables with *p* value less than 0.05 were retained for multivariate analysis. b ^1^: crude regression coefficient; b ^2^: adjusted regression coefficient. CI: Confidence interval. Partial eta squared: Estimated effect size. Bolded *p* values and partial eta squared indicate comparison inter-variables whereas un-bolded values were comparison groups within the same variable using least square difference (LSD).
